# Influence of High-Fat Diets Consumed During the Juvenile Period on Hippocampal Morphology and Function

**DOI:** 10.3389/fncel.2018.00439

**Published:** 2018-11-20

**Authors:** Nuria Del Olmo, Mariano Ruiz-Gayo

**Affiliations:** Departamento de Ciencias Farmacéuticas y de la Salud, Facultad de Farmacia, Universidad CEU-San Pablo, Madrid, Spain

**Keywords:** learning, memory, hippocampus, high-fat diets, obesity, leptin, fatty acids, PPAR

## Abstract

The negative impact of obesity on neurocognitive functioning is an issue of increasing clinical interest. Over the last decade, a number of studies have analyzed the influence of high-fat diets (HFDs) on cognitive performance, particularly in adolescent individuals. Different approaches, including behavioral, neurochemical, electrophysiological and morphological studies, have been developed to address the effect of HFDs on neural processes interfering with learning and memory skills in rodents. Many of the studies have focused on learning and memory related to the hippocampus and the mechanisms underlying these processes. The goal of the current review article is to highlight the relationship between hippocampal learning/memory deficits and nutritional/endocrine inputs derived from HFDs consumption, with a special emphasis on research showing the effect of HFDs intake during the juvenile period. We have also reviewed recent research regarding the effect of HFDs on hippocampal neurotransmission. An overview of research suggesting the involvement of fatty acid (FA) receptor-mediated signaling pathways in memory deficits triggered by HFDs is also provided. Finally, the role of leptin and HFD-evoked hyperleptinemia is discussed.

## Introduction

Elevated consumption of so-called western diets (WDs) is one of the main causes of overweight and obesity and a matter of concern for public health institutions. The damaging effect of these diets seems to be not only related to their content in terms of both saturated fat and easily assimilated carbohydrates, but also to the fact that they promote disorganized feeding patterns consisting of frequent energy-dense snacking and/or copious meals before bedtime (Corwin and Hajnal, 2005; Matheson et al., 2012, 2014). Closely related to this, the concept and term “comfort food” has been coined, referring to the consumption of palatable, high caloric food to mitigate stress and/or anxiety (Dallman et al., 2003).

The effect of WD within the brain is multifactorial since such diets promote changes in the blood-brain barrier (BBB) and choroid plexus permeability (Hsu and Kanoski, 2014; Hargrave et al., 2016), inflammation (Pistell et al., 2010), biochemical processes compatible with neurodegeneration (Kanoski and Davidson, 2011; Boitard et al., 2012), changes in both dopaminergic (Naneix et al., 2017; Romaní-Pérez et al., 2017) and glutamatergic neurotransmission (Valladolid-Acebes et al., 2012) as well as neurocognitive deficits (Noble and Kanoski, 2016). In this regard, a pivotal issue that deserves particular attention is that WD consumption itself, in the absence of obesity/overweight, has the capacity to cause neurocognitive impairment (Beilharz et al., 2015). This suggests that dietary components (a certain fatty acid (FA), a particular saturated/unsaturated FA ratio, the presence of elevated mono- and disaccharides, etc.) may be sufficient to cause neurocognitive effects—and that these effects are not only the result of metabolic impairment, such as brain insulin resistance, caused by the regular consumption of these diets (Bomfim et al., 2012; Derakhshan and Toth, 2013).

The concept of WD applied to animal research is confusing as this term covers diets with different qualitative and quantitative composition. The most commonly used WD are the so-called high-fat diets (HFDs; Van Heek et al., 1997), which yield mostly lipid-derived calories from saturated fat (lard), but also contain an elevated proportion of unsaturated fat and sucrose. The proportion of lard in these diets usually ranges from 2% (this diet is often used as a low-fat, control diet) to 32%, and the lower the amount of fat, the higher the amount of sucrose, with these two constituents ranging between 33% and 9%, respectively. Surwit diets, also used for this kind of study, contain hydrogenated coconut oil and corn-starch as fat and carbohydrate sources, respectively. Finally, the so-called cafeteria diets, containing elevated and undefined amounts of saturated fat and sugar have been also widely used to induce overweight/obesity. Hence, the complexity of all these diets makes it difficult to draw clear conclusions regarding the relative contribution of either fat or sugar to brain alterations. In this respect, exposure to sugar-sweetened beverages has been shown to have a negative impact on spatial memory (Reichelt et al., 2016) and neurogenesis (Reichelt et al., 2015), but systematic studies aimed at identifying the effect on the brain of either sugar- or fat-enriched diets are lacking.

As a result of all of the circumstances described above, comparison between different studies is very challenging and this might explain the discrepancies observed between such studies, despite them having been carried out under conditions that are apparently similar. Most studies reviewed in this article deal with the effect of van Heek-type diets.

## Unhealthy Diet has an Impact on Cognitive Performance: Epidemiological Features

The relationship between diet composition and cognitive impairment has been the focus of observational studies showing that improving dietary habits positively influences cognition (Drover et al., 2009; Spencer, 2010), while reduction in diet quality is associated with declining psychological functioning over the follow-up period (Jacka et al., 2011).

A number of clinical studies highlight that hippocampus-dependent declarative memory is damaged in overweight/obese adults (Nilsson and Nilsson, 2009), as well as in children/adolescents (Cserjési et al., 2007; Afzal and Gortmaker, 2015). Accordingly, an inverse correlation between body mass index (BMI) and academic performance has been established (Yau et al., 2014).

Related studies have revealed slower learning rates in hippocampus-related tasks in humans consuming high-sugar diets (Attuquayefio et al., 2016). Another clinical study revealed that a 4-day HFD reduces the extent of hippocampus-dependent learning and memory as well as interoceptive sensitivity (Attuquayefio et al., 2017). Some meta-analyses have also provided evidence for the negative impact of obesity on neurocognitive functioning (Liang et al., 2014). In addition, although the mechanism linking overweight/obesity and cognitive dysfunction remains poorly characterized (Sellbom and Gunstad, 2012), cumulative evidence has identified obesity as a risk factor for cognitive impairment (for a review see Castanon et al., 2014), including Alzheimer disease (De Felice and Ferreira, 2014).

Many studies have reported that memory impairment triggered by diets is associated to specific insulin resistance (Banks et al., 2012; Kim and Feldman, 2015). In this regard, a study in humans indicated that glucose, but not insulin fasting levels, may have an impact on episodic memory in middle-aged women (Backeström et al., 2015). Thus, understanding the mechanisms connecting the consumption of unhealthy diets and cognition deficits is becoming both urgent and necessary in order to develop effective strategies aimed at preventing the expanding global burden of co-morbid obesity and dementia.

## Consumption of High-Fat Diets During the Juvenile Period Impairs Hippocampus Morphology and Function

### Neurogenesis and Cell Morphology

The negative impact of HFDs on hippocampal neurogenesis has been the focus of many studies (Hwang et al., 2008; Park et al., 2010), some of which have shown a differential effect on animals that started to consume a HFD after weaning compared to those that consumed the diet during adulthood (Boitard et al., 2012).

Evidence that HFDs can affect neuron development is provided by other studies showing that obese young mice consuming these diets for 8 weeks display an unexpected increase in hippocampal spine density, accompanied by an up-regulation of the neural cell adhesion molecule (NCAM) expression in CA1 pyramidal neurons (Valladolid-Acebes et al., 2013). Similarly, another study has recently reported a similar effect within the prefrontal cortex in young rats undergoing prenatal exposure to HFD (Rincel et al., 2018). In contrast to these findings, Wang’s group (Wang et al., 2016) identified a decrease in hippocampal spine density in obese juvenile rats exposed to HFDs. Interestingly, a study by Ferreira’s group has reported that switching to a standard control diet reduces body weight (BW) and restores levels of hippocampal neurogenesis in these models (Boitard et al., 2016).

In addition to neuron morphology, astrocytes seem to be sensitive to HFD. This issue has mainly been investigated in hypothalamic areas (Chowen et al., 2016), but research specifically targeted within the hippocampus is less abundant. In this regard, a study carried out in HFD obese mice reported that consuming HFD from the time of weaning leads to longer and less abundant astrocyte prolongations (Cano et al., 2014) as well as to a reversible activation of hippocampal microglia (Hao et al., 2016). Obese adult rats undergoing a similar treatment displayed a lower number of GFAP-positive astrocytes (Gzielo et al., 2017), and a concomitant increase in the number of *Iba1* positive microglia cells was detected in non-obese mice subjected to a similar dietary intervention from weaning (Vinuesa et al., 2016).

### Effect of High-Fat Diets on Hippocampal Neurotransmission

It has been reported that HFD impairs synaptic efficacy and blunts *N*-methyl-D-aspartate (NMDA)-induced long-term depression (LTD), but does not affect long-term potentiation (LTP; Valladolid-Acebes et al., 2012), in the hippocampus of obese mice exposed to these diets during the adolescent period. These findings differ from those of other authors. For instance, Mielke’s group reported that HFD did not affect synaptic efficacy or LTP (Mielke et al., 2006), whereas other authors have demonstrated impairment of LTP after HFD treatment (Hao et al., 2016). The different periods of HFD treatment and the different protocols for synaptic plasticity processes may be responsible for these discrepancies. Moreover, Del Olmo’s group has identified the impairment of LTD as the most relevant change in synaptic plasticity due to HFD consumed during the juvenile period (Valladolid-Acebes et al., 2012). Other authors have observed that, in spite of an identical basal synaptic transmission, obese HFD-treated adult male—but not female—mice display lower LTP and LTD values compared with their respective controls (Hwang et al., 2010)—a result that paves the way for research related to gender-specific effects of HFD on hippocampal function. In addition, a 6-month HFD treatment has been shown to decrease basal synaptic transmission and LTP in the *dentate gyrus* of the adult rat hippocampus (Karimi et al., 2013).

These apparent alterations of hippocampus plasticity strongly suggest an influence of HFD on glutamatergic neurotransmission. In fact, a study carried out in obese mice that consumed HFD during the adolescent period, has shown that HFD improves glutamate (GLU) up-take kinetics along with the up-regulation of glial GLU transporters (GLT-1 and GLAST) and a concomitant down-regulation of glutamine synthase. In addition, this treatment led to the down-regulation of the glucose transporter GLUT-1 (Valladolid-Acebes et al., 2012). All of these findings suggest that HFD can trigger a dramatic de-regulation of GLU turnover and provides further support for the above-mentioned changes in hippocampus synaptic transmission and plasticity elicited by HFD interventions during the juvenile period. This and other studies have reported that post-weaning HFD down-regulates the expression levels of both the NR2B subunit of the NMDA receptor and synaptophysin, concomitantly with a detrimental cognitive impairment in rats (Page et al., 2014) and mice (Valladolid-Acebes et al., 2012).

GLU is not the only neurotransmitter that is sensitive to HFD within the hippocampus; GABA levels appear to be decreased in the hippocampus as well as in the prefrontal cortex of adult rats, and it has been proposed that this change might contribute to the disruption of GABAergic inhibitory processes and be related to changes in GLU metabolism (Sandoval-Salazar et al., 2016), which are coherent with the above-mentioned worsening of synaptic plasticity.

### Influence of High-Fat Diets During the Juvenile Period on Hippocampus-Dependent Spatial Memory

Research aimed at characterizing the effects of HFD consumption and/or HFD-induced obesity on learning and memory processes is abundant and has frequently been reviewed (Davidson et al., 2005; Cordner and Tamashiro, 2015; Noble and Kanoski, 2016). However, differences in the animal strain and age, the length of the dietary treatment and the experimental approach used all make it difficult to draw clear conclusions (Cordner and Tamashiro, 2015).

The effect of HFD on brain areas related to cognition, such as the amygdala, the prefrontal cortex, and especially the hippocampus, has received much attention in recent years. In the amygdala, Vega-Torres et al. (2018) recently reported that rats that consumed HFD exhibited attenuated fear learning associated to astrogliosis, which contrasts with the enhancement of emotional memory and amygdala plasticity reported by Boitard et al. (2015). Spencer et al. (2017) reported that HFD evokes neuroinflammation together with impaired amygdala-dependent memory. Janthakhin et al. (2017) also reported that maternal HFD impairs amygdala-dependent memory. Nevertheless, switching adolescent HFD to a control diet in adulthood reverses neurocognitive alterations (Boitard et al., 2016). With respect to the effect of HFD within the prefrontal cortex, a number of studies have implicated this brain area in cognitive deficits triggered by HFD (Reichelt et al., 2016), which may be related to morphological alterations evoked by these diets in this area (Dingess et al., 2017).

Although a study has reported that HFDs can have an influence in the ventral hippocampus of female mice (Krishna et al., 2015), the effect of HFD on memory seems to affect mainly dorsal hippocampus-dependent learning but spares other forms of learning (Stouffer et al., 2015). This may be due to the selective impairment of the dorsal hippocampus caused by oxidative stress, inflammation and/or disrupted neurotransmission produced by consumption of these diets. In this sense, HFD intake results in a negative influence on the hippocampus in terms of both spatial learning and reference memory, and it has been proposed that HFD triggers obesity in part by interfering with the inhibition of hippocampal-dependent memory, which is critical to adjust energy intake to meet energy demands (Davidson et al., 2007; Kanoski and Davidson, 2011). Most of the studies agree that HFDs impair hippocampal function. Thus, impaired hippocampus-specific spatial memory, evaluated in the radial maze paradigm, was detected in adult rats after 3–5 days on a high-fat/high-sugar diet, while no effect was observed in the case of hippocampus-independent memory tasks (Kanoski and Davidson, 2011). The impact of HFD on hippocampus-dependent learning and memory during the particularly vulnerable juvenile period has been assessed in a number of studies showing that these diets deteriorate both relational and spatial memory (Valladolid-Acebes et al., 2011, 2013; Boitard et al., 2012; Kaczmarczyk et al., 2013; Del Rio et al., 2016). This behavioral impairment is accompanied by the inhibition of hippocampal neurogenesis in mice (Boitard et al., 2012), as well as by morphological alterations of dendritic spines (Valladolid-Acebes et al., 2013). Moreover, HFD has been shown to evoke hippocampal inflammation in rats (Boitard et al., 2014). These observations do not appear to be linked to obesity, as Vinuesa’s group demonstrated that these processes are present without BW gain (Vinuesa et al., 2016).

Similarly, the effect of prenatal and perinatal exposure to HFD has also been shown to have a negative influence on the hippocampus in terms of spatial learning and reference memory (White et al., 2009; Lépinay et al., 2015; Wolfrum and Peleg-Raibstein, 2018). Related to this, a recent study carried out in humans demonstrated that 4 days of HFD reduces hippocampus-dependent learning and memory as well as interoceptive sensitivity (Attuquayefio et al., 2017). However, other studies carried out in adolescent mice have reported that short-term HFD fail to induce cognitive impairment in the novel object recognition paradigm (Del Rio et al., 2016).

The possibility that mood disorders triggered by obesity and/or HFD may have an impact on memory performance is a factor that has to be taken into account, since the increasing prevalence of childhood obesity has been accompanied by a parallel increase in comorbid psychological conditions such as depression and anxiety (Russell-Mayhew et al., 2012). Nevertheless, data in the literature do not allow the assessment of the comorbidity between memory and mood impairment. In this regard, some studies have reported anxiety and anhedonia after a 16-week HFD in adult rats (Dutheil et al., 2016) and after a 12-week treatment in mice (Sharma and Fulton, 2013), while other authors have reported a mood improvement together with a concomitant worsening of memory performance after 48 h HFD (Del Rio et al., 2016). The independence between mood and memory is stressed by the study by Kaczmarczyk et al. (2013), showing that learning/memory impairment evoked by HFD was not inhibited by the anti-depressants desipramine or reboxetine. For recent reviews on this topic, see Baker et al. (2017) and Reichelt and Rank (2017).

The influence of maternal obesity and high-fat feeding on synaptic plasticity, learning and memory has been also studied and recently reviewed by several authors (Contu and Hawkes, 2017; Edlow, 2017). Nevertheless, although there is compelling evidence of learning and memory deficits in the offspring on obese mothers (both in humans and in experimental models of diet-induced obesity), the mechanism that account for such an effect remains to be further investigated. As potential causes, a deficient production of BDNF within the hippocampus (Tozuka et al., 2010; Kim and Park, 2018), brain inflammation (Kang et al., 2014), and even a deficient leptin signaling (Dodds et al., 2011; Cordner and Tamashiro, 2015) may underlie deficient neurodevelopment and poor memory/learning performance observed in these models.

## Nutritional vs. Endocrine Inputs in Memory Impairment Triggered by High-Fat Diets

**Figure 1:**
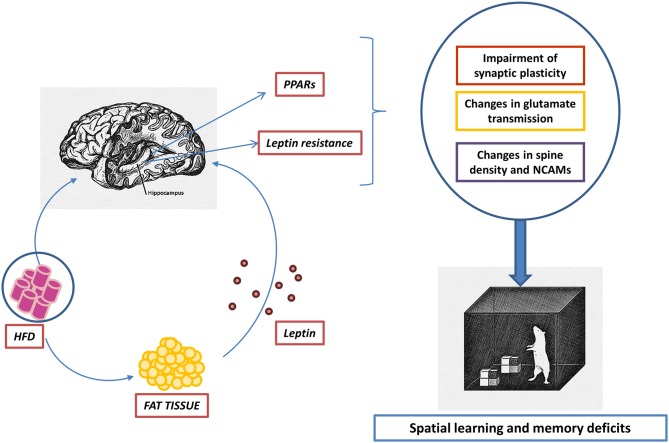
Hypothetical dual effect of high-fat diets (HFDs) on the hippocampus. Fatty acids (FAs) contained in HFDs enter the brain by crossing brain barriers. Elevated concentrations within the hippocampus of saturated FAs, such as palmitic acid, lead to a deficient activation of PPARγ together with an overstimulation of PPARα. The consequence of such an imbalance may be inflammation accompanied by a decrease of synaptic plasticity. On the other hand, HFDs trigger adipose tissue inflammation together with an altered pattern of adipokine secretion, characterized by an increase of circulating leptin. Hyperleptinemia causes leptin resistance able to limit the activity of signaling pathways relevant in maintaining the structural and functional integrity of the hippocampus.

Gut hormones and nutrients—which are pivotal for brain development and maturation—reach the brain after crossing the BBB and/or the choroid plexus by means of specific carriers as well as by unspecific diffusion mechanisms. These inputs can be altered by HFD as, in addition to an abundant supply of saturated FAs, these diets cause an imbalance of adipokines in white adipose tissue (WAT), characterized by the induction of leptin synthesis and the repression of adiponectin expression, which occur together with a rise in WAT-derived inflammatory cytokines, all of which are able to cross brain barriers (Tilg and Moschen, 2006; Spencer et al., 2017; Figure 1). In addition, HFDs have been shown to reduce the synthesis of bile acid receptor ligands, an event that may contribute to the impairment of neuroplasticity evoked by HFDs (Jena et al., 2018).

Systematic studies aimed at identifying the role of saturated fat as an independent risk factor for memory and mood impairment, specifically in the adolescent population, are lacking. A study by Baym et al. (2014) showed a negative correlation between hippocampus-dependent relational memory and the intake of saturated FAs in a child/adolescent population, supporting the concept that saturated fat impairs memory processes independently of metabolic factors. Nevertheless, the Baym study did not identify saturated fat intake as a BMI-independent risk factor for memory damage. Related to this, a study by Moon et al. (2014) demonstrated that acute administration of palmitic acid induces anxiety-like behavior in mice, independently of metabolic alterations.

The influence of HFDs on brain and particularly on hippocampus signaling, seems to involve many neurotransmitter (Hansen et al., 2018) and hormone-dependent pathways. In this context, insulin receptor signaling in hippocampal neurons is pivotal for spatial memory performance. The influence of HFD interventions on insulin responsiveness is difficult to determine, as most studies do not go beyond the identification of insulin resistance indexes (HOMA-IR), which are not indicative of the responsiveness of insulin receptors within brain areas involved in learning/memory. In this regard, the study by Vinuesa et al. (2016) reported that memory impairment triggered by HFD in juvenile mice is associated to specific insulin resistance within the hippocampus, even in the absence of obesity, as already observed by Mielke et al. (2006). These findings are in line with previous studies, carried out in engineered rats, showing that lentiviral deletion of insulin receptors within the hippocampus has a negative influence on hippocampus-dependent learning/memory tasks (Grillo et al., 2011, 2015). This suggests that integral downstream signaling of insulin receptors is pivotal for learning/memory performance. Otherwise, brain insulin resistance evoked by HFDs has been shown to impair hippocampal synaptic plasticity and memory by increasing the palmitoylation of the AMPA GLU subunit GluA1 (Spinelli et al., 2017). Memory deficits have also been observed—both in mouse and rat models, after 1- (Molteni et al., 2002) and 8-month dietary treatments (Stranahan et al., 2008)—associated to marked obesity and insulin resistance. Nevertheless, other studies have reported that consumption of HFD for 4 or 8 weeks, triggering BW increase but failing to alter insulin resistance indexes, evokes hippocampus-dependent memory deficits specifically in adolescent mice (Valladolid-Acebes et al., 2011). In humans, one study indicates that glucose—but not insulin fasting levels—may have an impact on episodic memory in middle-aged women (Backeström et al., 2015). In the same vein, hippocampus-dependent memory deficits in obese adult mice, exposed to HFD during the juvenile period, were not reversed by further limited access to HFD (Valladolid-Acebes et al., 2013). Nevertheless, other studies have reported that cognitive function is improved by subsequently switching to a standard chow (Woo et al., 2013; Boitard et al., 2016), and this reversibility also concerns other aspects such as neuronal plasticity (White et al., 2009; Lépinay et al., 2015), morphological changes (Rincel et al., 2018) and memory (Boitard et al., 2016). Taken together, all of these findings support the concept that it is diet composition—rather than obesity or elevated caloric intake—that is pivotal for the long-term effect of HFD on learning/memory.

All of this research points to uncertainty regarding the relative contribution of nutritional inputs vs. the influence of endocrine-metabolic impairment triggered by HFD. At this point, it should be highlighted that central inflammatory processes subsequent to HFD intake may be another key point to be considered. This topic has been the focus of recent reviews by Castanon et al. (2015), Morin et al. (2017), Spencer et al. (2017) and Layé et al. (2018).

## The Role of Saturated Fatty Acids in Memory and Learning Deficits: Facts and Hypothesis

FAs are not only able to generate cell components or precursor metabolites, but also bind both intracellular and cell surface receptors, including peroxisome proliferator-activated receptors (PPARs) and G protein-coupled receptor 120 (GPR120).

PPARs mediate pleiotropic actions in the brain, including neurogenesis and synaptic plasticity (Roy et al., 2016; Zhou et al., 2016), as well as physiopathological responses, such as inflammation (Luna-Medina et al., 2005). Although the implication of PPARs in neural processes involved in learning and memory has been analyzed in a number of studies, research specifically devoted to assessing the relevance of FA receptors in neural damage triggered by HFD in the juvenile brain is lacking. Nonetheless, the importance of these receptors in neurogenesis and inflammation processes clearly points to their involvement in long-term memory deficits elicited by juvenile HFD. The up-regulation of NCAMs has been demonstrated in CA1 pyramidal neurons, occurring concomitantly with an increase in spine density in mice exposed to HFD only during the adolescence period (Valladolid-Acebes et al., 2013), showing the importance of these diets in neurogenesis. As NCAM play key roles in learning, memory and synaptic plasticity (Becker et al., 1996), it could be hypothesized that the up-regulation of NCAM expression induced by HFD is an integral compensatory reorganization of CA1 neurons aimed at improving synaptic connectivity (Figure 1).

Saturated FAs are less efficient PPARα and PPARγ agonists than unsaturated FAs (Kliewer et al., 1997; Varga et al., 2011). Therefore, a deficient activation of PPARγ and a subsequent limiting of PPARγ-mediated functions could be expected from the intake of diets containing elevated saturated/unsaturated FA ratios, as occurs with HFDs. In this regard, palmitic acid has been shown to impair amyloid processing both in neurons and astrocytes (Patil et al., 2006), whereas pioglitazone (PPARγ agonist) improves learning in Alzheimer disease models (Papadopoulos et al., 2013). Other studies have reported that palmitic acid induces lipotoxicity in cortical rat astrocytes (Wong et al., 2014), reduces hippocampal neurogenesis (Park et al., 2011) and promotes inflammatory responses, which were absent after oleic acid administration (Gupta et al., 2012). Concerning the effect of these diets on PPARα activation, a recent study by Huang et al. (2017) reported a down-regulation of the astrocyte GLU transporter, GLT-1, triggered by palmitic acid and other PPARα agonists. This result would be consistent with the presence of PPARα in rodent hippocampus (Roy et al., 2014; Rivera et al., 2014) as well as with the proposed role of PPARα in modulating synaptic plasticity in hippocampal neurons, and therefore in memory and learning (Roy et al., 2013).

Regarding the influence of HFD on GPR120 activity, there are no studies in the literature. Nevertheless, GPR120 seems to mediate the effects of Ω-3 PUFA and to improve both glucose metabolism and insulin sensitivity (Milligan et al., 2017). In addition, GPR120 seems to be relevant in the regulation of appetite and mood anxiety (Auguste et al., 2016). One speculative possibility is that GPR120 is poorly activated in animals subjected to experimental HFD, which might contribute to the metabolic and mood disorders triggered by these diets.

## Leptin as a Key Factor for Memory Changes Evoked by High-Fat Diets in Juvenile Animals

The rise in plasma leptin levels during HFD interventions has a dual effect, since leptin enhances CA1 LTP in rats (Oomura et al., 2006) and has been shown to be necessary for both brain maturation and learning/memory consolidation (Morrison, 2009; Guo and Rahmouni, 2011), whereas leptin resistance evoked by hyperleptinemia appears to be associated with deficits in hippocampal-dependent behaviors (Van Doorn et al., 2017).

Behavioral effects of leptin involve hippocampus leptin receptors (Harvey et al., 2006), which modulate JAK/STAT3, PI3K/Akt, MAPK and calcineurin signaling pathways (Morrison, 2009). The relevance of these pathways for hippocampal-dependent memory/learning has been investigated and there is compelling evidence that JAK/STAT3 modulates synaptic plasticity (Nicolas et al., 2012). Moreover, PI3K/Akt and MAPK, and MAPK and calcineurin regulate LTP and LTD, respectively (Harvey, 2013). In support of this, Farr et al. (2006) have reported that leptin therapy improves cognitive deficits in adult mice displaying a spontaneous overproduction of amyloid precursor protein. Some authors have shown that *db/db* mice, which have an inactivating mutation in the leptin receptor, display cognitive deficits (Dinel et al., 2011). Conversely, delivery of leptin within the ventral region of the hippocampus suppressed conditioned place-preference for food, increased the latency to run for food in an operant runway, and suppressed memory consolidation in a non-spatial appetitive response paradigm (Kanoski and Davidson, 2010).

On the other hand, hyperleptinemia triggers a rapid desensitization of leptin transport mechanisms located within the BBB and the choroid plexus—an effect that limits the effect of leptin. Although this process seems to be reversible in adult animals (Banks and Farrell, 2003), little is known about the influence that juvenile obesity can have on brain barrier permeability to leptin in the adult brain.

Hyperleptinemia also impairs leptin receptor signaling both in neurons and glial cells in brain areas relevant for learning/memory (Grillo et al., 2011). It has been demonstrated that leptin resistance selectively affected the functionality of the PI3K/Akt signaling pathway (Valladolid-Acebes et al., 2013).

Interestingly, Mainardi et al. (2017) have reported that HFDs causes a loss of leptin-induced modulation of hippocampal synaptic transmission in mice.

## Concluding Remarks

Consumption of HFD during the juvenile/adolescent period has a negative impact on hippocampal memory and neural-related processes in the adult brain as revealed by a substantial amount of research carried out in murine models. Nevertheless, the variability of experimental conditions used to investigate this issue (dietary treatments of different duration, variety of diets, animals in different states of development, etc.) makes it difficult to draw reliable conclusions. Systematic studies carried out with diets of defined composition, which do not merge elevated amounts of saturated fat and sugar and that are preferably enriched in a particular FA, would be necessary to investigate in more depth the influence of fat on brain functions. In this regard, future research based on the use of experimental HFD manufactured with highly saturated or unsaturated oils, enriched in a particular FA, are needed to identify the specific contribution of the various types of dietary fat to memory processes.

Otherwise, the translational value of all of these findings remains unclear, as strong epidemiological studies are lacking. Therefore, it is necessary to carry out parallel clinical and basic research devoted to the identification of the molecular mechanism underlying memory deficits evoked by the regular consumption of HFD.

## Author Contributions

NDO and MR-G contributed to the conception and design of the review, as well as the drafting and revision of the manuscript. The authors have approved the final version of the manuscript.

## Conflict of Interest Statement

The authors declare that the research was conducted in the absence of any commercial or financial relationships that could be construed as a potential conflict of interest.

## Abbreviations

BBB: Blood-brain barrier
BMI: Body mass index
BW: Body weight
FA: Fatty acids
GLU: Glutamate
HFD: High-fat diet
LTD: Long-term depression
LTP: Long-term potentiation
NCAM: Neural cell adhesion molecule
NMDA: N-methyl-D-aspartate
WAT: White adipose tissue
WD: Western diet.
